# Research as an essentiality beyond one’s own competence: an interview study on frail older people's view of research

**DOI:** 10.1186/s40900-021-00333-7

**Published:** 2021-12-24

**Authors:** Maria Haak, Synneve Ivanoff, Emmelie Barenfeld, Isak Berge, Qarin Lood

**Affiliations:** 1 Research Platform for Collaboration for Health, Faculty of Health Science, Kristianstad University, Kristianstad, Sweden; 2 Department of Health and Rehabilitation, Institute of Neuroscience and Physiology, Sahlgrenska Academy, Centre for Ageing and Health – AgeCap, University of Gothenburg, Gothenburg, Sweden; 3 Department of Psychiatry and Neurochemistry, Institute of Neuroscience and Physiology, Sahlgrenska Academy, Centre for Ageing and Health – AgeCap, University of Gothenburg, Gothenburg, Sweden; 4 Institute of Health and Care Sciences, Sahlgrenska Academy, University of Gothenburg, Gothenburg, Sweden; 5 Centre for Person-Centred Care (GPCC), University of Gothenburg, Gothenburg, Sweden; 6 Centre for Ageing and Health – AgeCap, University of Gothenburg, Gothenburg, Sweden; 7 Department of Occupational Therapy and Physiotherapy, Sahlgrenska University Hospital, Gothenburg, Sweden; 8 School of Nursing and Midwifery, Faculty of Health Sciences, La Trobe University, Melbourne, Australia

**Keywords:** Frail older people, Patient and public involvement, Research, User involvement

## Abstract

**Background:**

There is an increased interest to make the voices of frail older people heard in research by actively involving them in research processes. Involving frail older people in research could, however, be perceived as challenging by researchers. To actively involve frail older people in research processes in a meaningful way, the knowledge about their own views on what research is must be widened and deepened.

**Methods:**

Individual interviews were conducted with 17 frail older men and women with former experience of participation in research studies. Qualitative data were analysed using content analysis.

**Results:**

Frail older people’s views on what research means are described through the main category; *An essentiality beyond one’s own competence,* which describes research as a complex process that is important for society but difficult to understand. This is described in the sub-categories; *A driving force for societal development, A benefit when based on lived experience, A source of knowledge difficult to access and understand,* and *A respected job filled with responsibilities.*

**Conclusion:**

Different views on research from the perspective of frail older people show that research is viewed as a complex yet important phenomenon to frail older people. Research was also seen as a natural part in society. Research was viewed as difficult to access and understand. Thus, researchers must train themselves to communicate research findings to the public in an understandable way. To create common understandings through information and education, researchers might be better placed to involve frail older people in a meaningful way and thereby also have the possibility to develop good working practice and relationships with those involved.

## Plain English summary

It is essential to the make voices of frail older people heard in research. Therefore, there is a growing interest to find ways to involve this group. However, to involve them in research is perceived as challenging for many researchers and frail older people might be excluded without acceptable reasons for exclusion. For frail older people to be involved in research processes, researchers must understand older peoples views, how to engage them and how to make research more inclusive. We therefore interviewed seventeen frail older people about their view of research, i.e., we asked questions on what research is and what it means for them. We found that research is viewed as important and necessary for societal development, but also as a something that is difficult to understand for the interviewed group. The participants did not feel competent or responsible for conducting research, and they respected researchers who they viewed as a group with great knowledge. Another pattern in the interviews was that the participants viewed research on frail older people’s everyday issues as important, and that this was an area where they could contribute. What we can learn from these descriptions is that it is important to strive for collaboration that attends to the different experiences and needs of frail older people and support them to understand research findings and make their voices heard in an allowing research environment.

## Introduction

Patient and public involvement in research has been described as a way to increase the benefits and relevance of research findings to different target groups. It is also believed to create deeper understandings of the investigated issues [1]. However, frail older people are an underrepresented group in research [2], both with and without patient and public involvement. Even though frail older people are a growing part of the population [3], there is a knowledge gap with regard to how to make use of their knowledge and experiences, and how they could contribute to research that may concern them. Frailty is commonly defined as living with an ageing, physically and physiologically declining body, which influences both stamina and endurance often measured by exhaustion, weakness, slowness, low physical activity and unintentional weight alongside morbidity and dependence in daily activities [4, 5]. Such declining functions have previously been used as exclusion criteria in research, and frail older people have generally been considered as sources of data, rather than as partners with valuable knowledge [6]. This raises serious questions with regard to both the generalisability of findings, and to the possibilities for frail older people to make their voices heard, to influence research, and to make use of research findings. Indeed, ageing and frailty may have a negative influence on people’s abilities, but there are also vast resources amongst the ageing population, concealed by stereotypic views of ageing and frailty.

Originating in the belief to conduct research *with* persons and not *on* persons [1, 7], patient and public involvement in research ranges from consulting different groups, to studies in which patients or the public lead the research project and the researchers work with or for them [8]. Perceptions about patient and public involvement in research have been investigated from different perspectives. For example, informal carers have expressed that their involvement in research projects generated personal benefits [9], and health professionals’ have described it as an ongoing process in adapting practice and research to facilitate collaboration and the ability to co-create knowledge [10]. When it comes to frail older people, there are few explorations of their experiences of being involved in research processes. One recent study [11] described their experiences of being involved in research projects as an unusual and challenging process. Moreover, it has also been found that researchers are uncomfortable with actively involving participants in the research process [12], which calls for further explorations and development of knowledge that could support patient and public involvement in research.

Involvement of frail older people in research could be a tool for a deeper understanding of the resources and needs of the ageing population, which, in turn, could be used to improve the quality of health and social care [13]. However, there is a lack of knowledge and theoretical support for when and how to involve frail older people in research. Creating a common understanding as a basis for working in partnership in research projects is essential to facilitate patient and public involvement [14]. Being aware of similarities and differences between researchers’ and frail older people’s views on research is key in establishing work in partnership and may bridge reported barriers to involvement in research among frail older people as reported in a study by Berge et al. [11]. The same study highlight the importance of research that is close to the everyday life of frail older people, and that can capture experiences and a willingness to contribute to something positive for oneself and for others. The researchers are experts in research and the frail older people are experts of their needs, perceived problems and goals. However, hitherto, very few studies on how older people view research have been published. In order to optimally involve frail older people in research, researchers need to understand how they view research. Driven by the purpose to explore patient and public involvement in research from the perspective of frail older people, this paper therefore aimed to explore the view of research from the perspective of frail older people themselves.

## Method

This study was conducted within the larger research program UserAge: understanding user participation in research on ageing and health [15], which focuses on patient and public involvement in research on ageing and health. UserAge targets several different categories; frail older people, relatives, and healthcare professionals, and user representatives are included in the research program to work actively together with the researchers during all phases of the research program. Due to the focus of this study, no user representative is involved as co-author. However, the user representatives contribute with continuous input to research questions, methodology, manuscript preparation, including co-authorship in other studies within the research program.

### Ethics

This study was conducted in accordance with the Helsinki Declaration and approved by the Regional Ethical Review Board in Gothenburg (T097-18). Information about the study was given in plain Swedish. Before the start of the interview the participants had time to read about the study and their participation and pose questions to the interviewer. Written informed consent was obtained from all participants before the start of each interview.

### Participants

The participants were people 75 years of age or older, who had all participated in a randomised controlled trial that aimed to evaluate comprehensive geriatric assessment in a hospital setting [13]. Striving for diversity in age, sex and cognitive status, living situation, dependency in activities of daily living and level of education, a total of 31 persons out of the 155 participants in the randomised controlled trial were assessed as eligible for participating in this study. Contact details to those persons were delivered by the researchers responsible for the randomised controlled trial, and the second, third and fourth authors of this study tried to contact potential participants at the hospital or by phone for those who had been discharged. A total of seven persons could not be reached, and seven declined to participate, which means that 17 out of the 31 eligible persons agreed to participate in the study. They were between 76 and 95 years of age (median 85), eight persons (47%) were women, 59% were living alone and all of them were assessed as physically frail using the FRESH screening instrument at the time of participation [16].

### Data collection and analysis

Data were collected between February 2018 and March 2019 by the second, third and fourth author who conducted in-depth interviews with the participants in the participants’ homes. All interviews started with the open question: “Can you please tell me what it was that made you choose to participate in a research study”, followed by questions to spark discussion on the participants’ view of research. The interviews lasted between 14 and 86 min, with a mean of 49 min. All the interviews were recorded digitally and transcribed verbatim.

The interviews were analysed iteratively with conventional qualitative content analyses [17]. The analysis was performed in Swedish to stay true to the essence of the data. To obtain a general sense of the whole and to become familiar with data, all authors read the transcripts several times and listened to the recordings. After this naive reading, the transcripts were read again by the first author who did a first preliminary coding. The codes and their contents were discussed, clarified, and agreed upon between the first, second and third author. Codes were then categorised based on interpretations of underlying meanings and discussed among all authors. In order to enhance trustworthiness, peer debriefing meetings with all co-authors were held during the analysis process [18]. Input to the emerging analysis were considered and integrated. Finally, the first author optimised the analyses, resulting in the final version of the findings.

## Findings

**Fig. 1:**
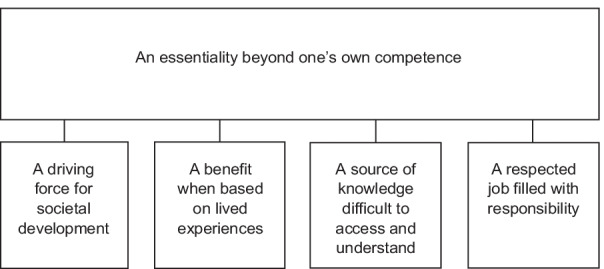
The overarching category and its sub-categories

The analysis resulted in one category and four sub-categories, describing the participants’ views on research as *an essentiality beyond one’s competence,* defined as: *A driving force for societal development, A benefit when based on lived experiences, A source of knowledge difficult to access and understand,* and *A respected job filled with responsibilities* (Fig. 1).

### An essentiality beyond one’s own competence

This overarching category describes the participants’ views of research as important and essential for development, while at the same time being complex and difficult to understand. Research was described as something that is being carried out by someone else, and was experienced as a process that exists outside one’s own area of responsibility and competence. Even though the participants described that they had some knowledge and understanding of what research is, they did not feel confident in what it is researchers do and why they do it. They did not experience themselves as aware of what is being researched, and they did not feel familiar with the research process. To elaborate on these experiences, the participants’ narrations are described in four sub-categories: *A driving force for societal development, A benefit when based on lived experiences, A source of knowledge difficult to access and understand,* and *A respected job filled with responsibilities.*

#### A driving force for societal development

This sub-category visualises research as a driving force for societal development. Research was described as a necessary process for societal progress. It was described as important and valuable, and as something that should be useful for the public. According to the participants, everyone in society should benefit from research, and research findings in general were perceived as significant for initiating societal development. Thus, research was an imperative contributor for a well-functioning society and there was an understanding that there is a continuous need for more research; the more research the better. To the participants, research also meant change and innovation, with researchers being qualified as persons who are always working on new research projects that contribute with opportunities for development. There was also an understanding of research as a lengthy process, and that the future will tell if research leads to development and change, visualised in the quotations:

In the future, it means that it will get better, it will improve. Isn’t that? what research is about?

The whole society is built upon research

#### A benefit when based on lived experiences

This sub-category reflects the understanding that, in order for research to be beneficial, it should emerge from a problem close to the person’s own everyday life or their surroundings. The experience was that research should target everyday problems, and on remedies and solutions that make everyday life work for ordinary people. That is, research should be conducted to facilitate everyday life for older people. The participants related to themselves and their experiences of what they had lived through when they expressed areas that they found important for research, and research that affected them as human beings was research on health and healthcare. Research with and for frail and vulnerable groups, and those who live in nursing homes was also highlighted as important, to increase these groups’ opportunities to live a good and dignified life. Moreover, research subjects close to the participants were pinpointed as especially important and necessary in order for research to be beneficial in terms of developing knowledge of relevance for older people. This was described by one of the participants as:

Yes, I have cancer myself so it is probably the kind of research I think you should spend more time and money on if you say so. I have many children and grandchildren and things might happen to them also, perhaps more research that would benefit them.

#### A source of knowledge difficult to access and understand

This sub-category illustrates that research was viewed as new knowledge, but only reachable for a limited audience. Accessing research and understanding research results was deemed difficult, and there was a large perceived distance between research, researchers and the public. The participants felt that it must be a challenge for researchers to communicate their findings in an understandable way. Thus, for research results to reach beyond the scientific community, the participants’ opinion was that it is necessary to disseminate research findings in forums where people affected by the research are located, and in a way that the public can understand. The participants did not feel that they were in control of how research was presented and how findings were disseminated, and they easily felt left out because research was difficult to comprehend. They wanted research to be presented in a clear and understandable way, as exemplified in the following quotation:

Very few read Illustrated Science (an easy to read popular science magazine)….many magazines… it is Greek to read...but if you could do something more for the same project, much like they do in Illustrated Science…that the public are able to read and absorb.

#### A respected job filled with responsibilities

This sub-category highlights that the participants had great respect for both research and researchers. Researchers’ efforts were highlighted as being above the “ordinary work of ordinary people”. Research was regarded as something that develops with life experience and was therefore not considered to be possible to carry out by everyone. According to the participants, research involved a constant learning process and there was a desire and a belief that researchers want to achieve and contribute to development and change rather than making money. Researchers were described as dedicated and stubborn people, with patience and curiosity. Being perceived as great thinkers who seek answers and take in different people’s perspectives, lifestyles and opinions, researchers were also considered to have great responsibility to do research on challenges relevant for the public and for society. As highlighted in the quote below, researchers were considered to have several responsibilities to consider in order to conduct research of high quality.

Researchers have a great responsibility to engage in the “right things”. And then there is the fact that research takes time. It must not go to fast. Then there is a risk that it is poorly substantiated.

## Discussion

This study offers insights in the view of research from the perspective of frail older people. Our main finding was that research was described as a complex yet important phenomenon, and as something that ought to have a natural part in society to contribute to development. However, research was also described as existing outside what frail older people regarded as their area of responsibility and competence.

To the best of our knowledge, no previous study has reported on frail older people’s view on research, and our study thus adds to the existing knowledge base. The two sub-categories *A driving force for societal development* and *A benefit based on lived experiences* jointly explain why and when research was experienced as valuable. The participants expressed research being valuable, important and necessary for societal progress. At the same time, research was expressed as valuable when beneficial for the individual as described in the category *A driving force for societal development.* This finding is comparable with statements of what research is, as it is described by the Swedish research council [19], being instrumental in development of individuals as well as societies. Further, in the category *A benefit when based on lived experiences* it was emphasised that for research to be beneficial for frail older people, research should be related to challenges faced by them as a group.

As described by Bratteteig and Wagner [20], patient and public involvement in research might also include a risk of power imbalance and this risk is even higher when frail older people are involved as their voices might be diminished by stereotypic views of ageing and frailty [21]. The participants of our study perceived research as inaccessible, which might create a distance between researchers and the public. Locock et al. [22], further report on how participants with experience of being actively involved in research projects might view themselves as outsiders, lacking expert knowledge and being the one's posing naïve questions. Experiences like these might lead to an imbalance in the relationship between involved partners. The phenomenon of power imbalances in research studies actively involving patients and the public is thus not new. Over the years, unequal power relationships in research studies involving researchers and non-academics have been highlighted in several studies [23–25]. In addition, unequal relationships have been found to be a barrier for user involvement [26]. In order bridge such barriers, previous research studies have underlined that researchers need to put emphasis on communication, information about the topic and aim of the study, and plan for enough time and resources to meet individual needs of those involved [13, 27]. Thus, interpreted in relation to previous research on how to involve vulnerable groups in research studies [28] the present findings suggest that researchers carefully anchor research questions and dissemination of research findings with the frail older people themselves.

The present findings also suggest that research was perceived as challenging, not understanding all of the information that had been given. Health literacy is the ability to handle information, including the comprehension of verbal and written health information. Low health-literacy skills can affect all age groups [29] but when we age, people become more vulnerable to inadequate health literacy [30]. One way to make research findings more understandable is to facilitate translation of research findings [31, 32]. To make research findings understandable and usable, it is important to know how to optimally translate and disseminate research. Several models of how to translate research findings are available and in a recent literature review Esmail et al. [33] conclude that it is only through conscious use of knowledge translation theories, models and frameworks that implementation of research findings can be implemented in healthcare contexts. For example, it has been emphasised that research information should be disseminated in different ways through different media and communication channels. If being successful in making research findings more understandable for frail older people, healthcare services that build on research have the potential to become more effective, thus leading to better utilisation of care and outcomes for the older population [34].

As visualised in the present findings, a possible barrier to involvement of frail older people in research is that they might view research as something that is conducted by others and that requires specific competences and that they had great respect for both research and researcher. Trusting the researcher and the research is considered an essential prerequisite for involvement in research [35, 36]. However, research has found that trust is a dynamic concept involving building a relationship and interacting in respectful ways, but this trust can easily be broken [37]. Thus, even though the importance of involving people outside academia in research concerning health service design has been emphasised [37], it might not come as a surprise that the participants of the present study were somewhat hesitant towards active involvement in research processes as they are not used to view themselves as partners in research studies. To clarify the added value of frail older people’s contribution to research, how and when to be involved and to strengthen frail older people’s role as research partners is therefore important tasks for researchers in addition to a mutually agreed relationship important for both parties, and for the conduct of the research.

Further, to be sensitive to frail older people’s individual needs, preferences and prerequisites for involvement in research studies has also been pointed out as important for researchers to consider [12]. Training programs at the start of involvement in research projects have been shown to help people outside academia to understand why their involvement is important and which options there are to work together with researchers (e.g., in design, data collection, interpretation and dissemination). People outside academia felt more confident and gained a better understanding of how to contribute with their own experiences [14]. In order to conduct high quality research that is beneficial in societal development, it is, however, imperative to start out from the target groups’ views on research.

### Limitations

In this study, some limitations should be considered. First, the interviewed frail older people in general had a positive attitude towards research and it is important to acknowledge that there might be more negative attitudes among frail older people in other cultural contexts. There might also be practical reasons that hinder frail older people from involvement in research, such as communication and cognitive difficulties, transportation issues and socio-economic factors [38]. Previous research [14] also shows that one incitement for participation in research is the possibility to access healthcare services otherwise not available and thus this could be one reason for a positive attitude. However, it should be noted that the participants in this study were recruited due to their participation in another research project and thus most likely other incentives for their participation. Further, the findings illustrate complexities that comes with research and a range of views and attitudes about research according to frail older people. Second, healthcare is raised as an important area for research with and for frail older people. One could assume that individual experiences govern which parts of healthcare research that is emphasised by the individuals as important to research. This could of course generate bias. However, that healthcare research is raised as an important area to research is not surprising since the participants were asked for participation within a healthcare context and also had former experience of participation within this context.

## Conclusion

What the present study adds to the understanding of involvement of patient and public involvement is knowledge on frail older people’s view of research. The participants trusted the intentions of the researcher and described society as dependent on research. At the same time, research was viewed as difficult to access and difficult to understand for the public. To facilitate access to research findings and to make research more understandable, more research is needed to explore how to create supportive environments based on competence and with access to the educational tools to meet the needs of people with lower health literacy. This requires the participation of frail older people in collaboration with the researchers to promote allowing environments where research issues can be discussed in a safe and secure environment.

Through the promotion of allowing environments adapted to frail older people, the findings add to the understanding of and deepen the knowledge about how to tackle power imbalances between frail older people and researchers to diminish experiences of research as something beyond frail older people’s competences. Unequal relationships are barriers for user involvement, and researchers need to put emphasis on communication, information about the topic and aim of the study, and plan for enough time and resources to meet individual needs of those involved. Researchers needs to carefully anchor research questions and dissemination of research findings with the frail older people themselves. To create common understandings of what research means to different partners and clarify expectations and roles in relation to frail older people’s experiences of involvement opportunities as a basis for collaboration might play an imperative role when to actively involve frail older people in research studies. Through mutually planned information and education researchers might be better placed to involve frail older people in a meaningful way and thereby also have the possibility to develop good working practice and relationships with those involved.

## Data Availability

The dataset generated analysed during the current study are not publicly available due to the information provided to the participants when obtaining their informed consent, stating that all attempts would be made to maintain confidentiality. De-identified data are, however, available upon reasonable request to enable review, and will be stored for 10 years at the University of Gothenburg. All data are covered by the Public Access to Information and Secrecy act (offentlighets- och sekretesslagen) and a confidentiality assessment (sekretessprövning) will be performed at each individual request. Permission from University of Gothenburg, the Institute of Neuroscience and Physiology, has to be obtained before data can be accessed.

## Abbreviations

GPCC: Gothenburg, Sweden; Centre for Person-Centred Care
UserAge: Understanding user participation in research on ageing and health
FORTE: The Swedish Research Council for Health, Working Life and Welfare
